# Torsion of a Large Myomatous Uterus Associated with Progressive Renal Failure and Paralytic Ileus in an 86-Year-Old Woman

**DOI:** 10.1155/2019/1601368

**Published:** 2019-11-03

**Authors:** Guiwen Wang, Hiroshi Ishikawa, Asuka Sato, Makio Shozu

**Affiliations:** Department of Reproductive Medicine, Graduate School of Medicine, Chiba University, Chiba 260-8670, Japan

## Abstract

Uterine torsion of a nongravid uterus is rare, and proper diagnosis is challenging. Herein, we report a case of torsion of a large myomatous uterus in an 86-year-old woman who was presented with progressive renal failure and paralytic ileus. She was presented with abdominal discomfort, loss of appetite, and oliguria. A large myomatous uterus with broad calcification was identified when she underwent surgery to repair an umbilical hernia one year before the symptoms developed. Computed tomography revealed that one year later, the myomatous uterus significantly increased in size and the calcified lesion of the fibroid was largely displaced. She was also presented with paralytic ileus, and her general condition progressively worsened. Her serum creatinine levels were increased (3.5 mg/dL) and hemoglobin levels were low (8.5 g/dL). Emergency laparotomy revealed that the uterus was rotated 360 degrees clockwise at the level of the isthmus. The uterus was discolored, appearing dark red, and accompanied by broad congestion, and the cervix was elongated. The patient's renal function and ileus recovered after a hysterectomy. In conclusion, torsion of a large myomatous uterus could become life-threatening in an oldest-old woman, and early release of the torsion is necessary to avoid serious complications.

## 1. Introduction

Uterine torsion, defined as rotation of the uterine body by more than 45 degrees around the long axis of the uterus, is a rare but serious condition [1]. Most of the reported cases of uterine torsion involve a gravid uterus; however, a large myomatous uterus is another potential cause for uterine torsion [2, 3]. The uterus in its normal state has little mobility, as it is firmly held by round ligaments, broad ligaments, and sacrouterine ligaments; however, a large, heavy uterus such as a gravid or myomatous uterus may rotate, exerting traction on the uterus.

The most common symptom in women with uterine torsion is abdominal pain, but the presentation is nonspecific and variable, and can include vaginal bleeding, gastrointestinal problems, and urinary discomfort [3]. In addition, the symptoms vary from mild to severe, and they have the potential to progress into a life-threatening condition.

Herein, we report a case of torsion of a large myomatous uterus in an 86-year-old woman who was presented with progressive renal dysfunction, severe anemia, and paralytic ileus.

## 2. Case Presentation

**Figure 1:**
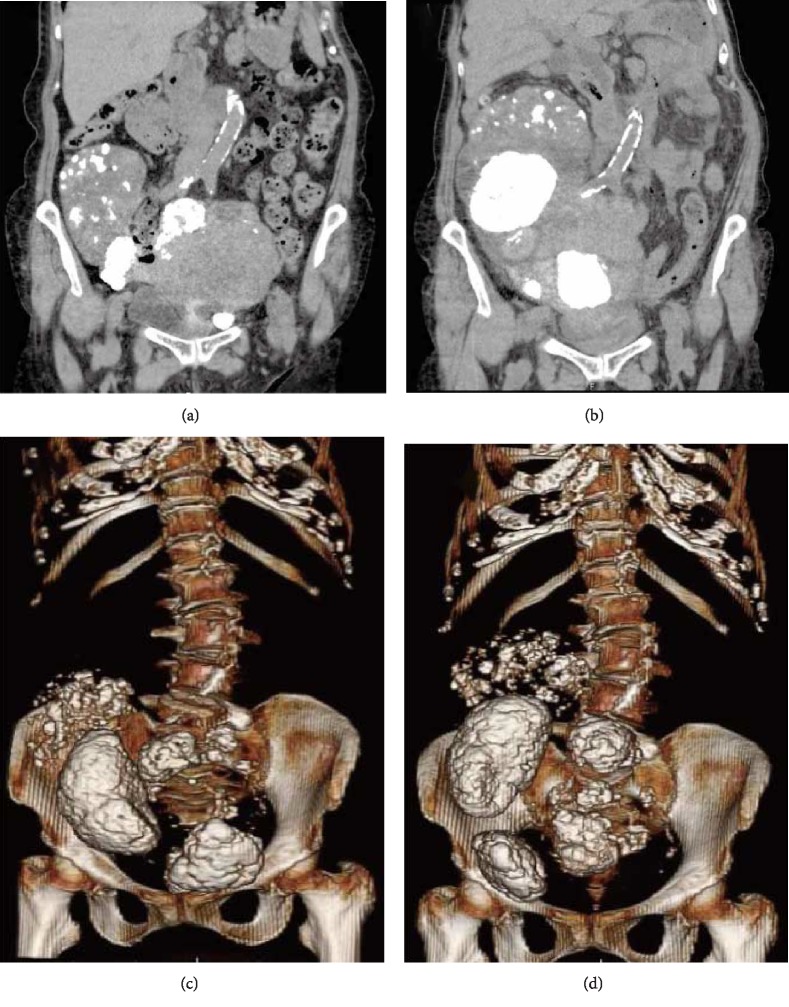
Computed tomography (CT) findings of the myomatous uterus. (a) Coronal section of the abdomen one year before the uterine torsion. The myomatous uterus and calcified portion are observed. (b) Coronal section of the abdomen at the time of uterine torsion. The myomatous uterus significantly increased in size, and the calcified lesion was displaced. (c) Three-dimensional reconstruction images of the lower abdomen one year before the uterine torsion. (d) Three-dimensional reconstruction images of the lower abdomen at the time of uterine torsion. The calcified area inside the uterus was largely displaced.

An 86-year-old woman (2 gravida, 2 para), who was presented with an increased pelvic mass, was referred to our facility. She had undergone surgery to repair an umbilical hernia one year previously, and a large abdominal mass with broad calcification, measuring 16 × 13 × 13.1 cm (longitudinal × transverse × anteroposterior), was identified by plain computed tomography (CT) before the hernia repair surgery. The mass was consistent with a uterus containing calcified uterine fibroids (Figure 1(a)). The patient felt the increased pelvic mass and experienced gastric discomfort related to the stomach being compressed by the mass, resulting in complete appetite loss for the previous week. She was also presented with continuous uterine bleeding and urinary discomfort. The latest CT examination showed that the myomatous uterus significantly increased in size, measuring 27 × 16.5 × 17 cm (Figure 1(b)), and the calcified lesion was largely displaced (Figures 1(c) and 1(d)).

**Figure 2:**
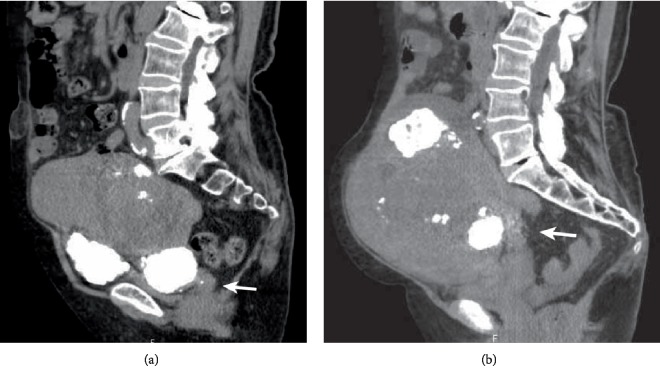
CT findings of the cervix. (a) Axial section of the lower abdomen one year before the uterine torsion. (b) Axial section of the lower abdomen at the time of uterine torsion. The cervix was elongated and moved upward compared with its position one year before (arrow).

On admission, her height was 150 cm and body weight was 60 kg. Her body temperature was 35.8°C, pulse rate was 81/min, and blood pressure was 88/40 mmHg. Although she was lucid, her Eastern Cooperative Oncology Group (ECOG) performance status was level 3, accompanied by disabling fatigue [4]. Her abdomen was distended by an irregular hard mass equivalent to 28 weeks' gestation. There was mild tenderness without rebound tenderness on the mass. On pelvic examination, the portio was normal and no vaginal bleeding was observed. Transabdominal ultrasound showed a large pelvic mass with peripheral calcification suggestive of uterine fibroids. Both ovaries were undetectable and minimal ascites was observed. Both kidneys were normal, with no dilatation of the renal pelvis and ureter. Plain CT images revealed that the uterine cervix was elongated and moved upward compared with its position one year before (Figure 2).

**Figure 3:**
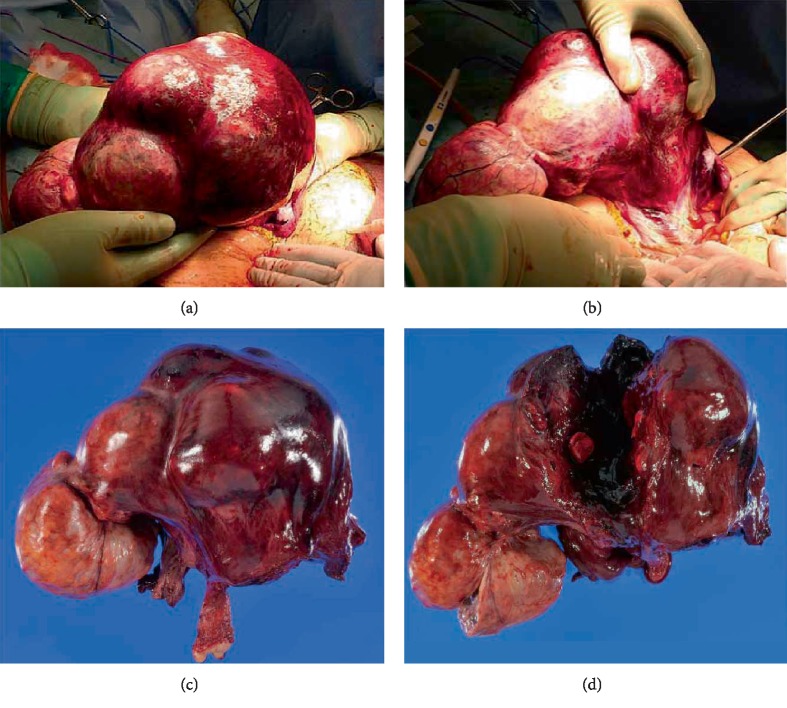
Operative findings of the twisted myomatous uterus. (a) The myomatous uterus was rotated 360 degrees clockwise on the long axis at the level of the isthmus. The uterine surface was discolored, appearing dark red because of the congestion. (b) After releasing the torsion, the uterine color turned red and the lower uterine segment was observed. (c) The resected myomatous uterus and bilateral adnexa. The surface of the uterus was dark red and the cervix was significantly elongated. (d) A cross-section of the uterus revealed the accumulation of clotting blood inside the uterus.

Blood tests showed an elevated white blood cell count (32,500/*μ*L), low hemoglobin levels (9.9 g/dL), elevated C-reactive protein (16.4 mg/dL), and high serum creatinine (2.45 mg/dL). We initially suspected a malignant tumor arising from the uterus or ovary, such as uterine leiomyosarcoma, carcinosarcoma, or ovarian cancer, based on the patient's advanced age, abnormal uterine bleeding, rapidly growing abdominal mass, and ascites. Therefore, we performed cervical cytology, endometrial cytology, and a puncture of the pouch of Douglas to assess the cytology of the ascites. The results of all cytological examinations were negative for malignancy. Transvaginal ultrasonography revealed that the endometrium was thin; therefore, we did not perform an endometrial biopsy. On the second hospital day, the patient's vital signs were stable, but her ECOG performance status fell to level 4. She was completely confined to the bed and could not carry out any self-care tasks; this change in status was accompanied by delirium. Paralytic ileus developed and her bowel was decompressed with a gastric tube. A small amount of uterine bleeding was observed, and her urine volume gradually decreased. Her hemoglobin level decreased (8.5 g/dL), and her white blood cell count (37,800/*μ*L) and serum creatinine levels (3.5 mg/dL) were elevated. On the third hospital day, she remained in a state of delirium. Hemorrhagic fluid was collected from the pouch of Douglas by a needle aspiration. The preoperative diagnosis was degeneration and infection of the large myomatous uterus; however, the relationship between the diagnosis and the deleterious change in her general condition remained unknown. During emergency surgery, the uterus was found to be rotated 360 degrees clockwise at the level of the isthmus (Figure 3). The rotated uterus had suffered total infarction. A small amount of hemorrhagic fluid was present in the peritoneal cavity. A part of the small intestine was compressed by the uterus. We performed a total abdominal hysterectomy and bilateral salpingo-oophorectomy. The weight of the uterus was 3.8 kg. The postoperative course was uneventful, and the patient was discharged on the seventh postoperative day. Histopathology showed fibroids and extensive uterine and adnexal hemorrhagic infarcts consistent with uterine torsion.

## 3. Discussion

In this report we describe an 86-year-old woman who experienced torsion of a large myomatous uterus. Torsion of a nongravid uterus is extremely rare, and most of the reported cases involve a large myomatous uterus [5–8]. Onset of uterine torsion can vary from reproductive age to oldest-old age. Although the mechanisms of uterine torsion are unknown, rotation could occur after the ligaments have been stretched for many years by the heavy myomatous uterus. The uterus may rotate due to its weight, thus exerting traction on the uterus. Other conditions reported in cases of torsion of a nongravid uterus are a normal-sized uterus accompanied by a large ovarian cyst [1, 9, 10] and a uterine anomaly in combination with complete cervical agenesis [11].

The most common symptom in women with uterine torsion is acute abdominal pain, but the symptoms and their severity are variable. A similar case of uterine torsion in an oldest-old woman has been reported, wherein the patient had a large myomatous uterus, bowel symptoms, and a rapid decline in hemoglobin levels [5]. She was presented with acute abdominal pain and distention for two days, but she had no history of vomiting, urinary symptoms, vaginal bleeding, or fever. However, our current case demonstrates that uterine torsion should still be considered even in the absence of severe abdominal pain.

Although our patient only presented with slight abdominal pain, and tenderness around the myomatous uterus was also mild, she presented with progressive renal dysfunction and severe anemia. Her general condition worsened progressively to a bedridden state. The mechanism of progressive renal dysfunction due to uterine torsion remains unknown. One possibility is a postrenal obstruction caused by deviation of the ureters. Torsion results in drastic elongation of the broad ligaments; this could obstruct the course of the retroperitoneal ureter at the level of the pelvic side wall. Another possibility is that severe congestion inside the rotated uterus may lead to acute cardiovascular failure accompanied by acute renal dysfunction. Progressive severe anemia is also associated with hypovolemia. Compared with reproductive-age women, oldest-old women are less adaptable during acute progressive hypovolemia.

Although preoperative diagnosis of uterine torsion is challenging because of its rarity and lack of specific symptoms, imaging findings are helpful for the diagnosis. Contrast-enhanced CT is one of the most valuable imaging methods for suspected uterine torsion. One of the characteristic findings is a whorled structure of the uterine cervix [5]. Multiplanar reformatted images are helpful for understanding the twisting of the cervix [8]. Both uterine torsion and pedunculated fibroid torsion show enhancement of the surrounding capsule of the uterine muscle layer and fibroid nodule. Unfortunately, it was impossible to perform contrast-enhanced CT for our patient because she was experiencing renal dysfunction; however, it was possible to compare the plain CT images between the onset of uterine torsion and images obtained one year previously. The notable imaging findings were that the volume of the myomatous uterus significantly increased in size and the cervix elongated and deviated upward at the onset of torsion compared with one year before. In addition, three-dimensional reconstruction of the images clearly showed substantial movement of the calcified lesion in the uterus. Given these results, we recommend CT imaging, ideally contrast-enhanced, for patients with a large, myomatous uterus after menopause to identify such changes and enable the early detection of uterine torsion. Congestion of the twisted uterus results in uterine enlargement accompanied by severe, progressive anemia. In general, uterine fibroids remain stable or decrease in size after menopause. If a myomatous uterus suddenly increases in size and the fibroid location changes after menopause, uterine torsion should be considered.

Differential diagnoses for torsion of a nongravid uterus include torsion of adnexal tumors, torsion of a pedunculated uterine fibroid, and infarction and/or degeneration of uterine fibroids. In cases of uterine torsion, a whorled structure of the uterine cervix on contrast-enhanced CT has been described. In cases of adnexal torsion, a thickened and twisted pedicle with adnexal tumors, primarily cystic tumors, has been identified. The twisted pedicle frequently contained the pedunculated, congested fallopian tubes. In cases of pedunculated subserosal uterine fibroids, most of the stalk is thin and usually undetectable by imaging. The pedunculated fibroid nodule is poorly enhanced. Magnetic resonance imaging (MRI) is useful for diagnosing the infarction and/or degeneration of uterine fibroids. Both intranodule hemorrhage and degeneration reveal high and low intensity mosaic patterns on T2-weighted images. Thus, imaging modalities such as contrast-enhanced CT and MRI can be used to properly distinguish between these conditions. It is noteworthy that torsion of a nongravid, normal-sized uterus and torsion of a large ovarian cyst have also been reported [9, 10].

## 4. Conclusions

Torsion of the nongravid uterus is an extremely rare condition, but it should be considered in menopausal oldest-old women with large uterine fibroids. Early release of the torsion may prevent progression to a critical condition caused by severe circulatory failure.
